# Modulating T Cell Phenotype and Function to Treat Hypertension

**DOI:** 10.34067/KID.0000000000000090

**Published:** 2023-03-23

**Authors:** Daniel J. Fehrenbach, Bianca Nguyen, Matthew R. Alexander, Meena S. Madhur

**Affiliations:** 1 Division of Clinical Pharmacology, Department of Medicine, Vanderbilt University Medical Center (VUMC), Nashville, Tennessee; 2 Department of Molecular Physiology and Biophysics, Vanderbilt University, Nashville, Tennessee; 3 Vanderbilt Institute for Infection, Immunology, and Inflammation, Nashville, Tennessee

**Keywords:** hypertension, basic science, checkpoint inhibitors, cytokines, hypertension, inflammation, interleukin 17A, T cells

## Abstract

Hypertension is the leading modifiable risk factor of worldwide morbidity and mortality because of its effects on cardiovascular and renal end-organ damage. Unfortunately, BP control is not sufficient to fully reduce the risks of hypertension, underscoring the need for novel therapies that address end-organ damage in hypertension. Over the past several decades, the link between immune activation and hypertension has been well established, but there are still no therapies for hypertension that specifically target the immune system. In this review, we describe the critical role played by T cells in hypertension and hypertensive end-organ damage and outline potential therapeutic targets to modulate T-cell phenotype and function in hypertension without causing global immunosuppression.

## Introduction

Hypertension affects nearly 50% of the US population and is the leading modifiable risk factor of worldwide morbidity and mortality because of its contribution to cardiovascular and kidney diseases, including stroke, myocardial infarction, heart failure, and renal failure.^1,2^ Approximately 50% of individuals with hypertension have poorly controlled BP, which is in part because of undertreatment.^3^ However, it is important to note that patients who are treated and are successful in achieving reasonable BP control still have an increased risk of cardiovascular events compared with their untreated counterparts with similar levels of BP,^4–6^ highlighting the critical need for novel therapies that address the underlying end-organ damage that accompanies hypertension. Immune cells have long been found in tissues from hypertensive animals and humans, but only recently have we begun to unravel the critical role played by these cells in promoting hypertension and hypertensive end-organ damage.^7,8^ Seminal studies demonstrated that an intact immune system is required for the full development of hypertension in mice and rats in response to multiple hypertensive stimuli.^9–12^ While these studies were conducted in rodents with deficiency of all lymphocytes, follow-up studies demonstrated that deletion of specific subsets of innate or adaptive immune cells or specific cytokines also resulted in blunted BP elevations and, more importantly, protection from hypertension-induced cardiovascular and renal injury.^7,8^ Thus, immune cells are key drivers of hypertension-induced end-organ damage.

Innate immune cells include dendritic cells (DCs), macrophages, and neutrophils. These cells are the first responders to injury or infection. Adaptive immune cells include T and B lymphocytes, which are recruited subsequently, and contribute to long-lasting immune memory. Numerous studies have linked T cells to hypertension, whereas the role of B cells is still unclear.^8,13^ T cells can be activated classically by antigen presentation from innate immune cells, particularly DCs. Kirabo *et al.* demonstrated that in response to hypertensive stimuli, DCs present a class of potential neoantigens composed of isolevuglandin (isoLG)-modified peptides that ultimately drive T-cell proliferation and proinflammatory cytokine production.^14^ Adoptive transfer of hypertensive DCs raised BP in wild-type recipient mice, but not in mice lacking lymphocytes, supporting the concept that DCs act through T cells to promote hypertension. IsoLGs form as a byproduct of oxidative stress. However, the precise isoLG-adducted neoantigen(s) formed in hypertension and whether these are unique to hypertension are unknown. Interestingly, T cells can also be activated in an antigen-independent manner by cytokines and other environmental stimuli, including salt and microbial metabolites.^15–17^ Thus, we propose that modulating T-cell phenotype and function is a potential therapeutic strategy to limit the end-organ damage associated with hypertension. Furthermore, it may be possible to do this in an antigen-agnostic manner and without causing severe immunosuppression.

In this review, we will first describe the different subsets of T cells, their functions, and characteristic cytokines. We will then discuss several signaling pathways within T cells that promote skewing of T cells into pro- or anti-inflammatory subsets and the known or potential effects of targeting those pathways on hypertension and hypertensive end-organ damage. Finally, we will discuss how a class of drugs that has revolutionized cancer therapy by activating T cells may have untoward consequences for the subsequent development of hypertension.

## T-Cell Subsets and Their Characteristic Cytokines

**Figure 1:**
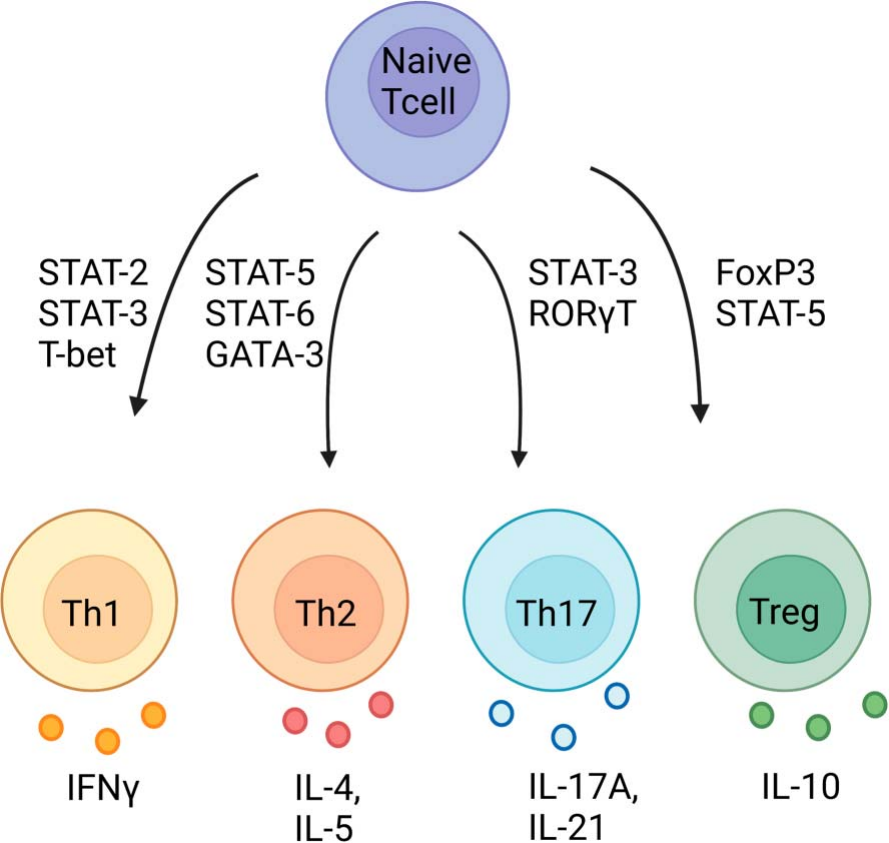
**T-cell subsets and their signature cytokines and transcription factors.** Naïve T helper cells use a combination of transcription factors shown here to differentiate into these major T helper cell subsets. FoxP3, forkhead box P3; GATA3, GATA binding protein 3; ROR*γ*T, retinoid orphan receptor gamma T; STAT, signal transducer and activator of transcription; T-bet, T-box expressed in T cells; Th1, T helper type 1; Th17, T helper 17; Th2, T helper type 2; Treg, T regulatory. Created with Biorender.

A critical role for T cells in experimental hypertension was established by several laboratories using immunodeficient mice and rats.^9,11,12^ T cells can be subdivided on the basis of their surface markers, cytokine profiles, and lineage-determining transcription factors, with each subset exhibiting precise functions in health and disease. Major T-cell classes are CD4^+^ T helper (Th) cells and CD8^+^ T cytotoxic (Tc) cells. CD4^+^ Th cells are further classified into Th1 cells that produce IFN*γ* and respond to intracellular pathogens, Th2 cells that produce IL-4 and IL-5 and are involved in allergic disorders and parasitic infections, Th17 cells that produce IL-17A and IL-21 and are involved in autoimmunity and the response to extracellular pathogens, and T regulatory cells (Tregs) that produce IL-10 and suppress immune responses (Figure 1). In addition to CD4^+^ Th and CD8^+^ Tc cells, a subset of innate-like lymphoid cells called gamma delta (γδ) T cells also make proinflammatory cytokines and play important roles in inflammation. We were the first to show that IL-17A, produced by Th17 cells and γδ T cells, plays a key role in hypertension and vascular dysfunction.^18^ Schiffren and colleagues demonstrated that depletion of γδ T cells alone protects mice from angiotensin (Ang) II-induced hypertension and vascular dysfunction.^19^ We and many others defined mechanisms by which IL-17A promotes endothelial dysfunction, vascular fibrosis, renal sodium retention, and renal injury.^20–24^ Recently, we found that IL-21 also plays an important role in hypertension and likely functions upstream of T-cell IL-17A and IFN*γ* secretion.^25^ Th cell production of IL-17A and IL-21 is increased in hypertensive animals and humans.^18,25^ Importantly, genetic deficiency or pharmacological depletion of IL-17A, IL-21, or IFN*γ* results in blunted hypertension and protection from end-organ damage.^22, 25–28^ By contrast, Treg cells play a protective role in hypertensive mouse models. Barhoumi *et al.* demonstrated that adoptive transfer of Tregs prevented Ang II–induced increases in BP, mesenteric artery endothelial dysfunction, and oxidative stress in the heart and aorta.^29^ At nearly the same time, Matrougui *et al.* showed that injection of Tregs from normotensive mice into hypertensive mice results in improved coronary arteriolar endothelium-dependent relaxation.^30^ Importantly, circulating Tregs are decreased in hypertensive humans.^31,32^ Whether boosting Treg numbers or function protects against hypertensive end-organ damage in humans is yet to be determined. CD8^+^ T cells can be similarly classified on the basis of cytokine secretion, and CD8^+^ T cells have been shown to play a critical role in hypertension.^33,34^

## SH2B3/LNK

**Figure 2:**
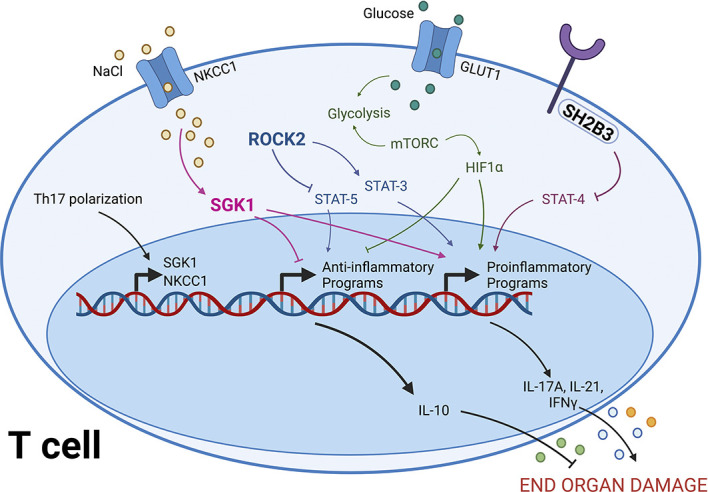
**Potential pathways to target to modulate T-cell phenotype in hypertension**. A T cell integrates various signaling pathways, which contribute to balancing the inflammatory phenotype. The T cell can respond to increases in NaCl by taking in NaCl through NKCC1, which is sensed as SGK1. SGK1 promotes proinflammatory signaling and inhibits inflammatory pathways. ROCK2 phosphorylates STAT3 and reduces phosphorylation of STAT5, which also shifts the cell toward an inflammatory phenotype. Inflammatory Th1, Th2, and particularly Th17 cells use glycolysis to generate energy. This is regulated by expression of Glut1 on the cell surface and mTORC activity. mTORC signaling involves HIF1α, a transcription factor promoting Th17 differentiation. SH2B3 serves as a negative regulator of STAT4 signaling, thus providing a brake on inflammatory activation. Modulating inflammatory balance by manipulating these pathways can influence the type of cytokine produced by T cells to affect damage on target organs. Glut1, glucose transporter 1; HIF1α, hypoxia-inducible factor α; mTORC, mechanistic target of rapamycin complex; NaCl, sodium chloride; NKCC1, sodium-potassium-2 chloride cotransporter; ROCK2, rho-associated coiled-coil containing protein kinase 2; SGK1, serum and glucocorticoid regulated kinase 1; SH2B3, SH2B adapter protein 3; STAT, signal transducer and activator of transcription. Created with Biorender.

Emerging data, initially from genetic studies in humans and later refined in animal models, demonstrate an important role for SH2B adapter protein 3 (SH2B3; also known as LNK) in regulating T-cell cytokine production in hypertension. SH2B3 is an intracellular adapter protein that negatively regulates cytokine and growth factor signaling.^35^ Multiple genome-wide association studies in humans identified a strong association between a missense polymorphism in *SH2B3*, rs3184504, and hypertension.^36–38^ Building on findings from these GWAS studies, Huan T *et al.* conducted a network analysis combining GWAS results with human whole-blood transcriptomic and molecular interaction networks. This unbiased analysis identified SH2B3 as a potential key driver of hypertension.^36^ Subsequent studies using mice with genomic excision of exons 2 through 6 of *Sh2b3* were performed by Saleh *et al.* to test whether Sh2b3 deficiency alters BP and end-organ damage in hypertension. Findings revealed that Sh2b3 deficiency increased BP, renal damage, and vascular dysfunction in response to angiotensin II.^26^ Importantly, bone marrow transplantation experiments revealed a major contribution of Sh2b3 in hematopoietic cells to limit BP elevation. Sh2b3 deficient mice also exhibited enhanced T-cell activation with increased IFN*γ* production in hypertension. Given that IFN*γ* deficiency reduced angiotensin II–induced BP elevations, these findings suggested that Sh2b3 reduces T-cell IFN*γ* production to limit BP elevations and end-organ damage in hypertension (Figure 2).^26^

Although findings described above demonstrate that loss of Sh2b3 in mice enhances hypertension severity, the relevance of these findings to humans harboring the rs3184504 polymorphism associated with hypertension remained unclear. Understanding the effect of this missense polymorphism became particularly relevant given studies in rats that a 6 bp deletion in the SH2 domain of Sh2b3 (distinct from the site of the rs3184504 polymorphism in the PH domain) resulted in *decreased* BP and renal inflammation, in contrast to findings from deletion of exons 3–6 in mice.^26,39^ Hence, our group used Crispr-Cas9 to generate mice homozygous for the major allele of this polymorphism encoding arginine (Arg/Arg) and mice homozygous for the minor allele encoding tryptophan (Trp/Trp) at the orthologous location of SH2B3 in humans. Results revealed that mice homozygous for the minor or risk allele (Trp/Trp) exhibited increased BP and enhanced renal damage compared with Arg/Arg controls. These mice also exhibited increased splenic and renal T-cell IFN*γ* production with hypertension induction and in response to IL-12 stimulation. These findings suggest that the rs3184504 minor allele is a causal variant for hypertension through at least a partial loss of function of Sh2b3 resulting in enhanced T-cell IFN*γ* production.^40^ Although Mori *et al.* demonstrated that Sh2b3 deficiency can have cell intrinsic effects in both DCs and T cells to promote IFN*γ* production,^41^ we did not find significant differences in dendritic and other myeloid cell cytokine production in mice with the Sh2b3 minor versus major alleles.^40^ Hence, it seems that Sh2b3 plays a particularly key role in limiting T-cell activation and cytokine production in hypertension.

Given multiple lines of evidence from humans and mice suggesting a key role for SH2B3 to limit BP elevations and end-organ damage in hypertension, SH2B3 represents a potential therapeutic target to modulate T-cell phenotype and hypertension severity. Although enhancing SH2B3 function would be expected to limit inflammation and reduce BP, therapeutic approaches to achieve this directly are challenging. Alternative approaches include inhibiting Jak-Stat signaling to limit T-cell activation, particularly Stat4, which promotes enhanced IFN*γ* expression and is activated to a greater degree in T cells harboring the *SH2B3* minor allele.^42^ This approach may be particularly efficacious in those of European ancestry who have a minor allele frequency for this variant of > 40%.^43^ Given that the *SH2B3* rs3184504 polymorphism is also associated with a variety of autoimmune diseases, including type 1 diabetes mellitus,^44^ rheumatoid arthritis,^45^ and celiac disease,^46^ identifying whether SH2B3 alters T-cell cytokine signaling in these conditions may also lead to new therapeutic approaches. Finally, SH2B3 is expressed in a variety of hematopoietic cells beyond lymphocytes and plays an important role in limiting signaling downstream of a variety of hematopoietic growth factors, including thrombopoietin, erythropoietin, and platelet-derived growth factor.^47,48^ Expression has also been described in endothelial cells where SH2B3 negatively regulates cytokine-induced apoptosis and adhesion molecule expression.^49–51^ Hence, systemic strategies targeting SH2B3 may also have broader effects on hematopoiesis and endothelial cell function.

## SGK1

There is a long-established relationship between dietary salt and hypertension because strict sodium homeostasis is required for normal physiological function in most organ systems. Sodium handling by the kidney is well-established, but there is a new appreciation for sodium sensing by other tissues, particularly immune cells. Excess salt has been linked to the development of inflammation and hypertension through upregulation of the salt-sensitive kinase, serum and glucocorticoid-regulated kinase 1 (SGK1). SGK1 is best known for its role in the kidney where it regulates surface expression of the epithelial sodium channel^52^ and activity of the renal outer medullary potassium channel.^53^ Mice lacking renal SGK1 have a salt-wasting phenotype,^54^ and SGK1^−/−^ mice are unable to appropriately excrete K^+^ in response to a K^+^ load.^55^ However, in addition to its effects in kidney tubule cells, there are emerging data that SGK1 in T cells independently promotes hypertension and hypertensive end-organ damage.

A balance between proinflammatory Th17 cells and Treg cells is critical for immune homeostasis and tolerance. SGK1 functions as a pivotal node in this balance by promoting the development of Th17 cells, particularly in response to salt, and restraining the development of Treg cells.^16,17,56^ Our laboratory investigated whether T-cell activation through SGK1 promoted hypertension. Th17 polarized cells exposed to an additional 40 mM of sodium chloride *in vitro* exhibited an upregulation of SGK1 and the IL-23 receptor, which is important in Th17 cell maintenance. This effect was abrogated by concurrent treatment with either furosemide or bumetanide (which inhibit the sodium potassium 2 chloride cotransporter 1 [NKCC1]) but not hydrochlorothiazide or spironolactone.^57^ This is evidence that T cells themselves are salt-sensitive and may use NKCC pathways to sense changes in salt. Although Na+ concentration in the blood is tightly regulated, recruitment to sites of hypertensive damage, such as the kidney, would expose T cells to elevated Na+ concentrations and promote inflammatory activation. Other work has shown that the skin may serve as an additional storage compartment for sodium,^58^ although this has recently been called into question by Rossitto *et al.*^59^ Another potential site where Th17 cells may come into a high-salt environment is in the gut.

Using mice with conditional deletion of SGK1 in T cells, we showed that SGK1 in T cells is necessary for the full development of a hypertensive phenotype in both angiotensin II and DOCA-salt models of hypertension (Figure 2). Furthermore, T-cell SGK1 deficient mice were protected from aortic and kidney immune cell infiltration, renal damage, and vascular dysfunction.^57^ A subsequent study demonstrated that female ovariectomized Wistar rats, which are salt-sensitive, exhibited increased NKCC1 expression in peripheral blood mononuclear cells in response to a high-salt diet.^60^ Du *et al.*^61^ showed an increase in SGK1 in hypertensive mouse spleen and that treating mice with an SGK1 inhibitor significantly reversed cardiac and renal dysfunction.

## ROCK2

Like SGK1, recent evidence has shown that rho-associated coiled-coil containing protein kinase 2 (ROCK2) is an enzyme that facilitates differentiation of naïve CD4+ T cells into Th17 cells and inhibits differentiation into Treg cells. Biswas *et al.* demonstrated that ROCK2 pathway activation was associated with production of Th17-associated cytokines, IL-17A and IL-21, and further showed that ROCK2 was responsible for Th17 activation through studies using dominant negative retroviral transduction *in vitro* and in ROCK2^+/−^ haploinsufficient mice. These studies also demonstrated that ROCK2 promotes phosphorylation of IRF4, a key transcription factor for Th17 differentiation.^62^ ROCK2 functions as a molecular switch in T cells by promoting phosphorylation of STAT3, a key transcription factor that directly enhances transcription of genes involved in Th17 differentiation, and inhibits phosphorylation of STAT5, a transcription factor that promotes Treg gene expression.^63,64^ ROCK activation of Th17 programs has been shown to be ROCK2-specific and does not involve the alternative isoform ROCK1 (Figure 2).^64^

Because ROCK2 is a key regulator of Th17 differentiation, other investigators have dissected its role in Th17-associated autoimmune diseases. As previously reviewed, IL-17A is a key driver of psoriasis.^65^ Studies using a novel ROCK2 inhibitor, KD025, have been shown to lower circulating levels of IL-17A and IL-23 and reduce symptom severity scores in patients with psoriasis.^66^ In a similar manner, ROCK2 activation has been implicated in rheumatoid arthritis,^67^ systemic lupus erythematosus,^68^ and inflammatory bowel disease.^69^ Excitingly, the experimental drug KD025, a ROCK2-specific inhibitor, received US Food and Drug Administration approval for the treatment of specific patient populations with graft-versus-host disease (GVHD) under the generic name belumosudil and the brand name Rezurock.^70^ Studies leading to this approval demonstrated that KD025 treatment ameliorated pulmonary dysfunction and collagen deposition in the lungs in a model of major MHC mismatch multiorgan GVHD and pathological scoring in a model of minor MHC mismatch sclerodermatosis GVHD.^71^ A follow-up phase II trial in human subjects showed clinically significant improvements in GVHD severity in patients treated with this ROCK2 inhibitor.^72^

We and many others have shown that hypertension, similar to the abovementioned autoimmune diseases, is associated with Th17 activation. Hence, it would follow that ROCK2 activation may play a role in the development of hypertensive pathology. ROCK activity in peripheral leukocytes correlates with systolic BP and cardiovascular risk.^73^ In addition, a study in human hypertensive and normotensive subjects showed that the pan-ROCK inhibitor Fasudil decreases peripheral vascular resistance.^74^ Decreases in BP were not observed in this study most likely because of acute administration over a short 15-minute infusion. We propose that ROCK2 activation may contribute to hypertensive pathology through chronic mechanisms including immune activation, regulation of renal function, and possibly direct effects on vascular function. Indeed, a number of studies showed that Fasudil greatly reduces vascular resistance and may be effective for the treatment of pulmonary and portal hypertension.^75,76^ However, no human studies have investigated ROCK2-specific inhibition in the pathogenesis of arterial hypertension. To underscore the need for specificity, a recent study using cardiac tissue-specific ROCK deletion demonstrated that ROCK1 is critical for protection from the effects of pressure overload in the heart. By contrast, these investigators found that ROCK2 promotes reactive oxygen species production, fibrosis, and cardiac hypertrophy.^77^ Owing to these opposing roles, ROCK2-specific inhibition may be necessary for beneficial cardiovascular effects in the context of hypertension and warrants further investigation.

## T-Cell Metabolism

T-cell subtypes use different intrinsic metabolic programs to execute their functions. Understanding how T cells create and use energy stores will not only help understand how and why T-cell activation is occurring in the context of hypertension but may also provide new therapeutic targets for modulating T-cell phenotype. It is important to note that cells will not exclusively use one particular mode of metabolism but may preferentially use one method over another because it contributes to the particular function of the cell.

Within CD4^+^ Th cells, there seems to be a delineation between Th1, Th2, and Th17 effector subtypes, which require glucose metabolism, and Treg suppressive cells, which use lipid oxidation.^78^ Activation of mammalian target of rapamycin (mTOR) signaling is required for T effector subtypes to maintain energy utilization and inhibition of mTOR induces a state of anergy.^79^ In addition, rapamycin (mTOR inhibitor) treatment prevents Th17 differentiation in response to IL-6 and TGF*β* stimulation and instead promotes Treg polarization.^80^ This has been confirmed using mTOR deficient T cells, which are unable to initiate Th1, Th2, and Th17 responses on appropriate stimulation. Not only are mTOR deficient T cells able to adopt a Treg phenotype, but they also exhibit enhanced suppressor activity and hypersensitivity to TGF*β* stimulation.^81^ For Th17 cells, HIF1α (a Th17-specific transcription factor) activation requires mTOR signaling to activate glycolytic pathways.^82^ In addition, HIF1α signaling has been shown to induce targeting of FoxP3 for proteosomal degradation, which further contributes to shifting Th17/Treg balance toward a proinflammatory state.^83^

Metabolic fuel can enter the cell through many pathways, including the SLC2 family of glucose and polyol transporters.^84^ Within this family, GLUT1 was the first to be identified^85^ and supports T-cell viability.^86^ Glut1 is critical for early development of T cells in the thymus and is essential for the functions of CD4^+^ T effector cells.^87–89^ Glut1 deficiency does not affect Treg suppressive capacity.^90^ This highlights the preference for aerobic metabolism and implies that glucose is not the primary way Tregs produce energy. In contrast to T effector cells, Treg polarization is promoted through fatty acid oxidation.^91–93^ Although not as extensively studied as CD4^+^ cells, CD8^+^ T cells also require HIF1α to induce glucose uptake and glycolysis to execute their functions.^94^ In the resolution phase of an immune response, CD8^+^ T cells go through a shift from using glycolytic metabolism to fatty acid oxidation, mirroring a shift from effector functions to memory functions. This shift is TRAF-6–dependent because TRAF-6 deficient CD8^+^ T cells are unable to sustain antigen-specific cells weeks after an initial immunization.^95^ Additional evidence for a required shift in metabolism has been shown with rapamycin treatment during the contraction phase of an immune response, which accelerated the generation of antigen-specific memory CD8^+^ T cells.^96^

The ability to target immunometabolism for therapeutic purposes in the context of hypertension is still in its infancy. Two recent studies by Dr. Cowley's group showed that treatment with the mTORC1 inhibitor rapamycin or the mTORC2 inhibitor PP242 is sufficient to not only prevent but treat salt-sensitive hypertension in the Dahl SS rat. Importantly, inflammation and renal damage were attenuated in these mTOR inhibitor-treated animals. As outlined, mTOR signaling is required for T-cell activation, and therefore, some of the beneficial effects observed may be due to an immunometabolic shift in T cells (Figure 2).^97,98^

## Immune Checkpoint Inhibitors

The rapid explosion in use of immunotherapies for the treatment of cancer may provide a unique opportunity to better understand inflammation and immune cell activation in hypertension. Immune checkpoint inhibitors (ICIs) have revolutionized the landscape of cancer therapy, improving patient prognosis across numerous cancer types. However, the efficacy of these therapies is limited by immune-related adverse events (IRAEs), which often require treatment discontinuation. ICIs, such as ipilimumab and nivolumab, work by targeting immune checkpoint receptors cytotoxic T-lymphocyte–associated protein 4 (CTLA-4) and programmed death 1 (PD-1), respectively. These checkpoint receptors, which are exploited by tumors to create an immunosuppressive microenvironment, work by dampening prolonged immune responses and promoting T-cell exhaustion. Blocking this inhibitory signal allows T cells to mount an effective antitumor response. However, these immune checkpoints also play a pivotal role in maintaining self-tolerance, and their blockade can result in aberrant immune responses resulting in a plethora of IRAEs, including increased cardiovascular toxicity. Notably, patients with cancer approximately 2 years after ICI therapy have a four- to seven-fold increased incidence of myocardial infarction and stroke.^99^ However, it remains unclear whether increased BP and/or hypertension incidence contributes to these increased cardiovascular events. A recent meta-analysis of randomized ICI clinical trials in cancer reported a modest increase in hypertension-related adverse events in patients receiving combined ICI and chemotherapy; however, another meta-analysis reported no change in hypertension-related adverse events.^100,101^ These studies were unfortunately unable to directly assess effects of ICI therapy on changes in BP in individuals with hypertension, so further study in patients with cancer on ICI therapy with long-term follow-up will be needed to determine effects of immune cell activation with these drugs on hypertension. Of note, a recent study by Turker *et al.* demonstrated that dual ICI therapy targeting PD-1 and CTLA-4 is associated with increased BP in patients with melanoma after 2 years of follow-up.^102^

**Figure 3:**
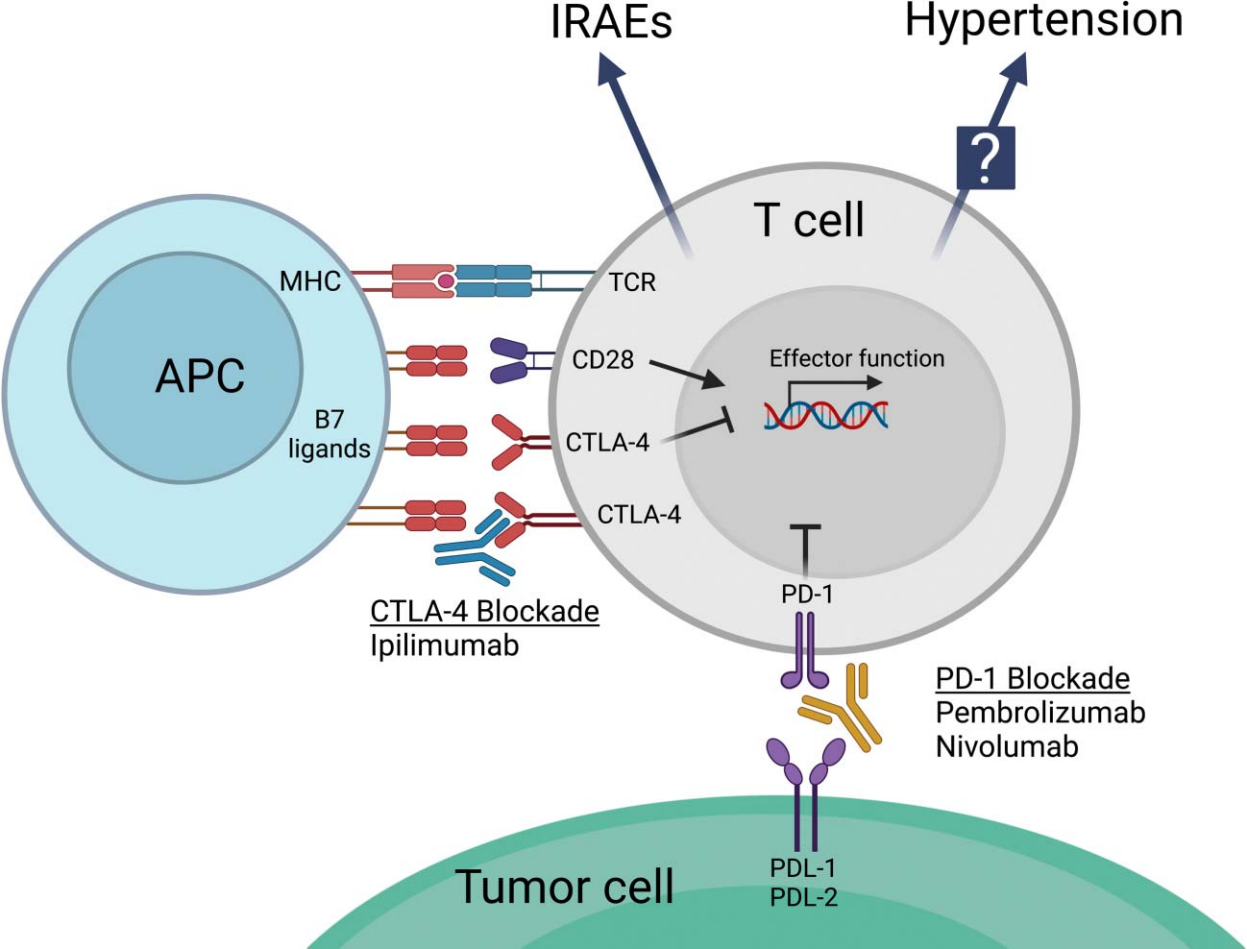
**CTLA-4 and PD-1 blockades promote T-cell activation to enhance antitumor responses but also promote immune-related adverse events (IRAEs) and may alter hypertension severity.** Because CTLA-4 competes with the costimulatory molecule CD28 for B7 ligands CD80 and CD86, CTLA-4 blockade enhances costimulatory B7 and CD28 interactions and limits T-cell inhibitory signaling by CTLA-4. PD-1 blockade also limits inhibitory signals from PD-1 interaction with ligands PDL-1 and PDL-2 to promote T-cell activation. APC, antigen-presenting cell; CTLA-4, cytotoxic T-lymphocyte–associated protein 4; PD-1, programmed cell death protein 1; PDL-1, programmed cell death ligand 1; PDL-2, programmed cell death ligand 2. Created with Biorender.

The mechanisms by which ICIs reinvigorate immune cells through blockade of their immune checkpoints suggest a potential benefit to agonizing these receptors to limit T-cell activation in hypertension. Full activation of T cells requires both engagement of the T-cell receptor (TCR) with the antigen presented on a major histocompatibility complex (MHC) and a costimulatory signal composed of binding of CD28 on the T cell with B7 ligands on the antigen-presenting cell. CTLA-4 is an effective negative regulator of this response by competing with CD28 for B7 ligands, for which CTLA-4 has higher affinity and avidity (Figure 3).^103^ Vinh *et al.* reported that interruption of this costimulatory signal could inhibit the inflammatory processes that underlie hypertension and vascular pathology. When treated with abatacept, a CTLA4-Ig, mice were resistant to angiotensin II–induced hypertension and vascular superoxide production. Mice treated with abatacept also had less circulating activated T cells and less immune infiltration into periadventitial fat.^104^ These findings establish T-cell costimulation as a necessary driver of vascular inflammation and represent a possible mechanism by which blockade of CTLA-4 may enhance cardiovascular inflammation, leading to hypertension.

PD-1 is essential for maintaining peripheral tolerance and keeping immune responses in an optimal physiologic range. PD-1 is expressed on T cells following activation and forms a negative feedback loop through the tyrosine phosphatase SHP2, which deactivates signaling elements of the TCR to reduce T-cell activation.^5^ Interestingly however, a recent report by Benson and colleagues demonstrated that PDL-1 in distal convoluted tubule cells induces salt retention in mice resulting in an elevation of BP. This salt retention occurred through enhanced interactions of PDL-1 on distal convoluted tubule cells with PD-1 on CD8^+^ T cells with resultant increased IFN*γ* production.^28^ Thus, the PD-1/PD-L1 pathway may have distinct, context-dependent effects on inflammation and salt retention in the context of hypertension. Also of note, PD-1 is expressed on immunosuppressive regulatory T cells (Tregs) and limits Treg function, so agonizing PD-1 may reduce inflammation through effects on conventional T cells but enhance inflammation through inhibition of immunosuppressive function in Tregs.^105^

Elucidating the cell-specific effects of ICIs will be key in determining their role and therapeutic potential in hypertension. Understanding the interplay of the mechanisms of immune checkpoints in cancer and cardiovascular disease has the potential to reveal novel mechanisms that may be targeted to modulate T cells as a unique and more effective treatment modality for hypertension.

## Conclusions and Future Directions

Initial elevations in BP due to primary hypertensive stimuli may lead to moderate tissue damage in target organs, such as the kidney and vasculature. Low-grade inflammation from this damage may lead to production of damage-associated molecular patterns, innate immune activation, and presentation of damage-related antigens. Preclinical studies, as outlined in this review, have shown that an adaptive T-cell response propagates the inflammatory response, amplifying tissue damage and potentiating hypertension. The changes in T-cell phenotype and T-cell cytokine production seen in experimental animals is also mirrored in human blood samples. Identification of hypertension-specific antigen(s) may provide a way to induce tolerance and avoid excessive immune activation. However, even without knowledge of any hypertension-related antigens, we propose that it may be possible to modulate T-cell phenotype and function for the treatment of hypertension-induced end-organ damage. In this review, we discuss several potential targets within T cells, including SH2B3/LNK, SGK1, ROCK2, immunometabolic pathways, and checkpoint inhibitors, that could potentially be exploited to modulate T-cell phenotype and function for the treatment of hypertension and related cardiovascular diseases. Some of these have effects in animal models of hypertension (*e.g.* SH2B3/LNK, SGK1, and checkpoint inhibitors) while others have not been extensively studied in hypertension specifically, but are predicted to play an important role on the basis of their known effects on T-cell function. Further studies are needed in animal models and humans to determine the ideal target(s) to limit hypertensive end-organ damage with minimal effects on immunosuppression. With advances in gene editing and nanoparticle delivery, perhaps T-cell–specific phenotypic modulation can be added to our future armamentarium of therapeutics to reduce morbidity and mortality from this worldwide silent killer—hypertension.
